# Opposite T_3_ Response of ACTG1–FOS Subnetwork Differentiate Tailfin Fate in Xenopus Tadpole and Post-hatching Axolotl

**DOI:** 10.3389/fendo.2019.00194

**Published:** 2019-04-02

**Authors:** Gwenneg Kerdivel, Corinne Blugeon, Cédric Fund, Muriel Rigolet, Laurent M. Sachs, Nicolas Buisine

**Affiliations:** ^1^Unité Mixte de Recherche 7221, Centre National de la Recherche Scientifique, Alliance Sorbonne Université, Muséum National d'Histoire Naturelle, Paris, France; ^2^Genomic Facility, CNRS, INSERM, Institut de Biologie de l'Ecole Normale Supérieure, Ecole Normale Supérieure, PSL Université Paris, Paris, France

**Keywords:** Thyroid hormone, Axolotl, network biology, embryonic development, paedomorphosis

## Abstract

Amphibian post-embryonic development and Thyroid Hormones (TH) signaling are deeply and intimately connected. In anuran amphibians, TH induce the spectacular and complex process known as metamorphosis. In paedomorphic salamanders, at similar development time, raising levels of TH fail to induce proper metamorphosis, as many “larval” tissues (e.g., gills, tailfin) are maintained. Why does the same evolutionary conserved signaling pathway leads to alternative phenotypes? We used a combination of developmental endocrinology, functional genomics and network biology to compare the transcriptional response of tailfin to TH, in the post-hatching paedormorphic Axolotl salamander and *Xenopus* tadpoles. We also provide a technological framework that efficiently reduces large lists of regulated genes down to a few genes of interest, which is well-suited to dissect endocrine regulations. We first show that Axolotl tailfin undergoes a strong and robust TH-dependent transcriptional response at post embryonic transition, despite the lack of visible anatomical changes. We next show that Fos and Actg1, which structure a single and dense subnetwork of cellular sensors and regulators, display opposite regulation between the two species. We finally show that TH treatments and natural variations of TH levels follow similar transcriptional dynamics. We suggest that, at the molecular level, tailfin fate correlates with the alternative transcriptional states of an fos-actg1 sub-network, which also includes transcription factors and regulators of cell fate. We propose that this subnetwork is one of the molecular switches governing the initiation of distinct TH responses, with transcriptional programs conducting alternative tailfin fate (maintenance vs. resorption) 2 weeks post-hatching.

## Introduction

Thyroid Hormones (TH) play central roles in numerous physiological and cellular processes, such as metabolism, cell proliferation, cell death, cell differentiation, and control of homeostasis. It is striking that a single hormone/signaling pathway can mediate such evolutionary conserved but functionally diverse transcriptional responses. TH actions are mediated through binding to specific receptors, the thyroid hormone receptors (THR), that belong to the superfamily of nuclear receptor transcription factors (1). Ligand binding modulates the receptor's biological activity, resulting in the transcriptional regulation of a large set of target genes (2, 3). TH stimulation typically leads to massive changes in the transcriptional state of the cell through both direct and indirect effects (2). To this respect, by offering a highly contrasted biological response, the Xenopus model has been instrumental to decipher the mechanisms of action of TH at physiological, cellular and molecular levels (3–6). The repertoire of TH target genes differs considerably in a cell- and/or tissue-specific manner, as it reflects new differentiation stages, metabolic states or other cell specific programs (7). Nonetheless, a very small number of genes (among which *klf9*) are differentially regulated in almost all tissues, suggesting that they belong to a core set of genes mediating TH response [reviewed in (7)]. Cross species comparison revealed that despite important species-specific TH responsive gene sets, homologous tissues often respond by using a core subset of genes (typically <1% of regulated genes), further strengthening the view of an evolutionary conserved molecular machinery (7). As such, and given its highly contrasted phenotypic changes, post-embryonic development has been a leading model to dissect the molecular, cellular and physiological changes and tissue remodeling initiated by TH signaling (8–10).

Post-embryonic development corresponds to the transition to a phenotypically distinct juvenile. This is an ancestral character shared by all extant tetrapods, and all chordates. Despite similar control by TH, the post-embryonic transition is quite diverse as it coincides with hatching in reptiles and birds (11, 12) and the perinatal period in mammals (10, 13, 14). In some species, this transition is so spectacular that it has been named “metamorphosis,” to reflect the profound morphological and ecological differences between a larva and a juvenile. In flatfish, post-embryonic development culminates with the migration through the skull of one eye of the symmetric larvae to the other side of the face (15). In anuran amphibians, this transition corresponds to the transformation of a tadpole into a frog (16). Most of the current knowledge on amphibian metamorphosis results from work on the anuran *Xenopus laevis* and *Xenopus tropicalis*, which have been instrumental in dissecting the anatomical, cellular and molecular processes taking place. In particular, the molecular mechanisms of the TH signaling have been the focus of numerous studies and are now well-described [reviewed in (3) and (17)], although this is still a subject of active research (18).

However, not all amphibians metamorphose and alternative post-embryonic development strategies are common, as seen in the case of paedomorphosis, where sexually mature “adults” of a species retain larval features of their ancestors (e.g., Ambystomatids) (8, 19). This relaxes the dependence on the changes of ecological niche imposed by metamorphosis (19). These alternative developmental strategies are thus ideal “natural experiments” for (1) investigating the link between TH signaling and the control of post-embryonic development, and (2) identifying the changes of transcriptional states driving the adaptive variations of post-embryonic transitions.

**Figure 1:**
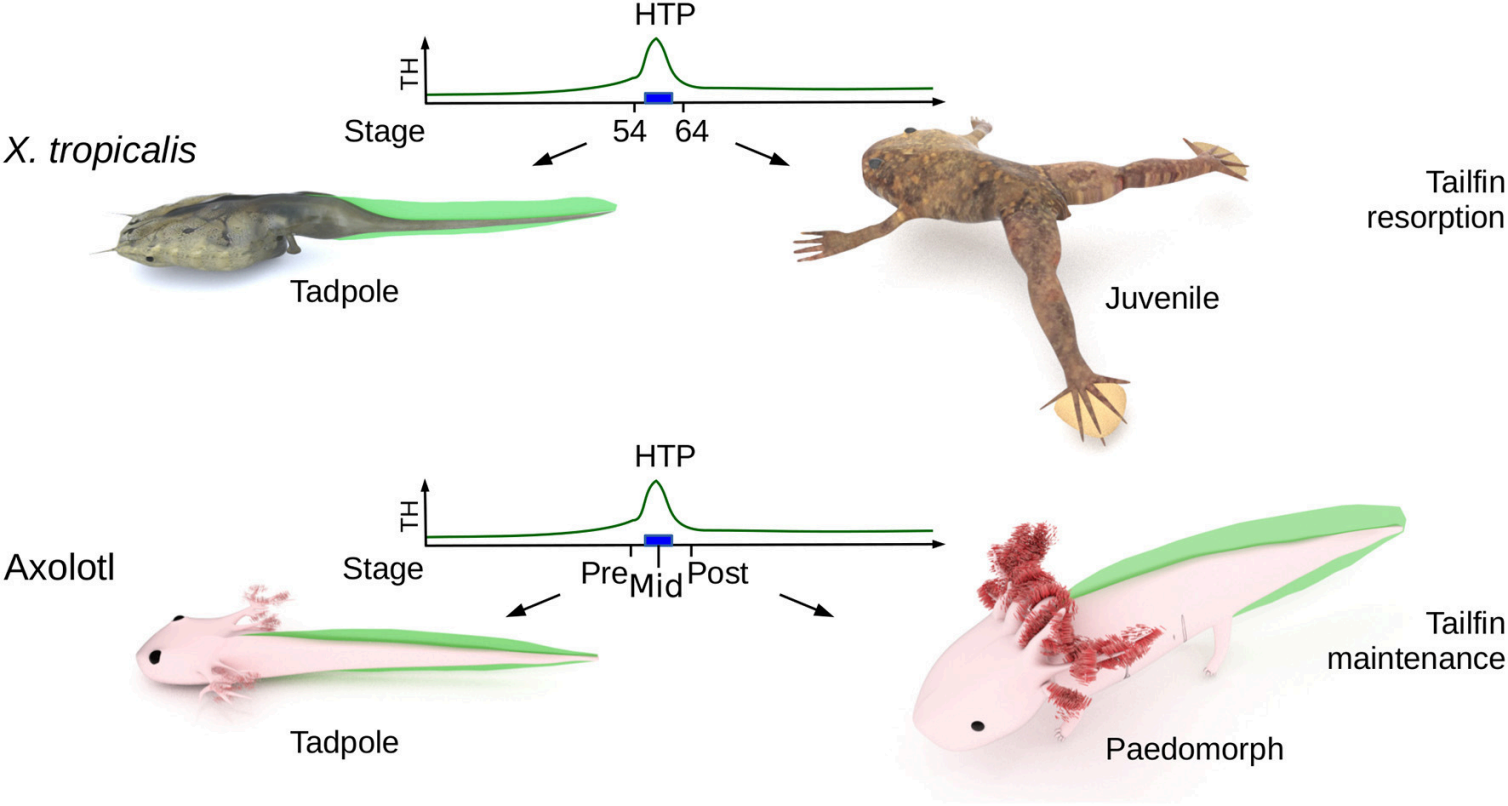
Biological context. The High Thyroid Hormone Period (HTP), that marks the end of tadpole stage (blue rectangle) in *X. tropicalis*, ignites metamorphosis and the resorption of larval tissues (such as tailfin). In Axolotl, the HTP does not induces tailfin resorption. “pre-HTP” animals will refer to class 3 Axolotl [as defined by Rosenkilde et al. (23)], when limb buds start growing (around 2 weeks post-hatching). At this stage, endogenous TH level is low and thyroid gland starts releasing TH. “mid-HTP” animals refer to class 8 Axolotl, with four toes on hind limbs (between 32 and 48 days post-hatching). This stage corresponds to the highest level of endogenous T_4_. “post-HTP” animals refer to class 12 Axolotl (around 3 months old), where T_4_ endogenous level dropped significantly. *X. tropicalis* tadpoles were staged according to the normal table of *Xenopus laevis* (Daudin) of Nieuwkoop and Faber (24). The TH levels are schematized from data of Leloup and Buscaglia (25) for Xenopus and of Rosenkilde et al. (23) for Axolotl. Digital paintings were carried out with BLENDER v2.8b.

The Mexican Axolotl (*Ambystoma mexicanum*) is the textbook example of an amphibian paedomorphic species (19) and is an attractive model to address these issues. Although rare in nature, metamorphosis in sexually mature Axolotl can be induced after long treatments with TH (20). Axolotl expresses functional TH receptors and typical TH response genes are differentially regulated [e.g., collagenase 3 and matrix metallopeptidase 11 (*mmp11*)] upon TH treatment in paedomorphs (21). As soon as 2 weeks post-hatching, the thyroid gland fixes iodine (22) and starts releasing TH, resulting in a peak of 20–40 nM thyroxine (T_4_) secretion [(23), Figure 1, developmental period later designated as the High TH Period (HTP)], but while limbs develop, larvae fail to metamorphose, as illustrated by other features such as gill growth and tailfin that remain anatomically unaltered (Figure 1). This TH peak also correlates with a rapid and transient increase of deiodinase2 (D2, TH activating enzyme) protein content in brain, up to the larvae-paedomorph transition, where closely related *Ambystoma* species undergo metamorphosis (but Axolotl doesn't). From this stage, D2 levels drop and deiodinase 3 levels (D3, TH inactivating enzyme), which were low, start raising (23). In agreement with these observations, early treatment of TH in rearing water prior to the endogenous TH peak does not induce metamorphosis (22), but results in miniature paedomorphs displaying extended growth of limbs and gills, but no tailfin resorption. Although previous report (26) describes accelerated development and early gills regression after intramuscular injection of TH in animals of various age (in the perivitelline space for 3 days post-spawning animals), this certainly does not reflect a physiological response because of the very high doses injected (10 to 60 μg TH per animal) and the fact that most animals died afterward. Therefore, it is not clear whether the lack of metamorphosis results from defect in TH signaling, as hypothesized previously based on experiments carried out on old paedomorphs (27). To date, the molecular determinism controlling paedomorphosis is still unknown and the current perception is that apart from the gonadal development program, paedomorph and larval tissues would be indistinguishable.

In this paper, we hypothesized that Axolotl tailfin almost certainly responds to TH, at the HTP. We ask whether and how Axolotl tailfin responds to TH at HTP. We also compared TH response between the Axolotl and the anuran amphibian *Xenopus tropicalis* (*X. tropicalis*), for which the post-hatching HTP is marked by metamorphosis (limb growth, and gills and tailfin resorption). We found that Axolotl tailfin responds strongly (>400 genes) and quickly (within 24 h) to TH. In the two species, tailfin response to TH mobilizes overlapping networks of biological pathways, despite little overlap between the two sets of differentially expressed (DE) genes. The developmental program (maintenance vs. resorption) is mirrored by alternative transcriptional states of a single subnetwork, structured around actg1 and fos. We thus propose that this subnetwork would be a component of a molecular switch involved in tailfin fate at HTP and the commitment into resorption vs. maintenance programs.

## Materials and Methods

### Animal Care and Treatments

All *Ambystoma mexicanum* (Axolotl) animals used in this study were obtained from Maison de l'eau (Villerville, France) and from a generous gift of Yannick Andéol (Sorbonne Université, Paris, France). Embryos were kept in tap dechlorinated water at room temperature and fed daily with artemia. They were staged with respect to the development table proposed by Rosenkilde et al. (23). Experiments have been conducted at class 3 (corresponding to 32 days old post-fertilization, around 2 weeks post-hatching, and prior to the TH peak during post-embryonic development), on class 8 (corresponding to 48 days old post-fertilization, corresponding to 1 month post-hatching, and at TH peak during post-embryonic development) and at class 12–13 (corresponding to 6 months old post-fertilization). We refer to these stages as pre-, mid- and post-HTP, respectively (Figure 1). The main active TH, T_3_ (T2752, SIGMA, St. Quentin Fallavier, France) was dissolved in 0.1 N NaOH and added to the culture medium to a final concentration of 10 nM. Control treatments correspond to an equivalent amount of 0.1 N NaOH. *X. tropicalis* tadpoles were raised at 26°C and staged according to the normal table of *Xenopus laevis* (Daudin) of Nieuwkoop and Faber (24). For TH treatment, tadpoles at stage NF54 were exposed 24 h to 10 nM T_3_. Animals were euthanized after anesthesia (ref: E10505; 0.01% MS222, SIGMA) before dissection. Animal care and experimental work was carried out in accordance with institutional and national guidelines and under permission granted in animal license number 00372.02 (*A. mexicanum*) and 68008 (*X. tropicalis*) delivered by the Cuvier Ethic Committee.

### RNA Extraction

For Axolotl, tailfin was collected from 5 individuals (8 to 11 groups per conditions) for pre-HTP (class 3) animals, from 5 individuals (3 groups per conditions) for mid-HTP (class 8) animals, from 3 individuals (5 groups per conditions) for post-HTP (class 12–13) animals and from 5 individuals (8 to 11 groups per conditions) for 6 months old paedomorphs. Tissues were flash frozen, and stored at −80°C before RNA extraction. For *X. tropicalis*, tailfins were collected from 10 tadpoles (3 to 8 groups per treatment conditions), flash frozen, and stored at −80°C. Tissues were lysed in 500 μl of RNAble (GEXEXT00-0U; Eurobio, Les Ulis, France) with one bead (INOX AISI 304 grade 100 AFBMA) using the Tissue Lyser II apparatus (QIAGEN, Courtaboeuf, France) for 1 min at 30 Hz. A total of 100 μL of chloroform was then added to the lysate. After 5 min incubation on ice, they were centrifuged 15 min at 12,000 g, 4°C. RNAs were purified from supernatant with the RNeasy MinElute Cleanup kit (ref: 74204, QIAGEN) according to the manufacturer's instructions. RNA concentration was measured with nanodrop and RNA quality was assayed using Agilent Bionalyzer with standard procedure. To avoid any potential contamination with genomic DNA, RNA samples were treated with DNAse following the provider instruction (Turbo DNA free; Ambion, Applied Biosystems, Courtaboeuf, France).

### Illumina Sequencing

Library preparation and Illumina sequencing were performed at the Paris Genomic Center (France). Messenger (polyA+) RNAs were purified from 1 μg of total RNA using oligo(dT). Libraries were prepared using the strand non-specific RNA-Seq library preparation TruSeq RNA Sample Prep v2 kit (Illumina). Libraries were multiplexed by 2 on a single flow cell lane and subjected to 100 bp paired read sequencing on a HiSeq 1500 device. For the comparison between T_3_ response at pre-HTP and 6 months old paedomorphs, libraries were multiplexed on 4 lanes on a NextSeq 5000 apparatus. Reads qualities were assessed with the FASTQC toolkit v0.11.3 (http://www.bioinformatics.babraham.ac.uk/projects/fastqc/). Axolotl raw data have been deposited on the Short Read Archive under accession numbers SRP067617, SRR810197, and SRR8101977, and X*. tropicalis* under accession numbers PRJNA240154. For the comparison between pre-HTP and 6 months old paedormorphs, samples were prepared following the same protocol, but subject to conventional 75 bp Illumina single end sequencing (TrueSeq), according to the manufacturer recommendations.

### ONT Sequencing

RNA samples were sequenced with Oxford Nanopore Technology, following standard procedures on two 1D^2^ flow cells, and raw data deposited under the SRA reference PRJNA498010.

### Transcripts *de novo* Assembly and Evaluation of the Assembly Procedure

Sequences were trimmed with AlienTrimmer v0.4.0 (28) to remove adaptor contaminants and low-quality sequences (Phred score ≤ 26). *De novo* assembly was carried out with Trinity v2.0.6 (29, 30) with ≥120 M paired-reads, and contigs shorter than 200 bases were discarded. Assembly with increasing number of paired-reads (5 to 120 M) randomly picked from the initial dataset showed that saturation is reached as early as 50 M reads from tailfin RNA. TRINITY run time options have little effect on assembly statistics. See (31) for a detailed description of the assembly and clustering procedures. The “long” reads produced by ONT sequencing of the same RNA samples were used to assess transcripts chimerism (Supplementary Figure 1). Assembly statistics are provided Supplementary Tables 1–3.

### Annotation of Transcriptomes Assembled *de novo*

Assembled reference sequences were aligned to the non-redundant (nr) protein sequence database (NCBI, release date November 2014) and to all *X. tropicalis* RefSeq protein sequences using BLASTX (32). BLASTX alignments took 720 days (CPU time) on a server with 16 cores and 64 GB of memory. Matches with a bit-score lower than 100 were discarded. Reference sequences were named according to the best reverse BLAST hit (BRBH, highest score). If several reference sequences were assigned to the same protein sequence, only the longest with the best score was kept. Annotation of Axolotl transcripts was carried out with the BLOSUM45 matrix.

### Differential Analysis of Gene Expression

For each species, filtered reads were mapped to the corresponding set of reference sequences with Bowtie v0.12.7 (33) and the “-l 32−3 10−5 10 -n 2 -m 1” parameters. Read counts were calculated for each reference sequences matched by at least one read and reference sequences with low read-counts (< 50) were discarded. To further estimate the accuracy of our differential expression analysis procedure in reflecting true biological variations, we compared the *X. tropicalis* gene expression levels to those obtained after conventional RNA-Seq carried out by our group (three biological replicates, single-end reads mapped on the genome sequence, unpublished data). We found that the log ratios from the two approaches correlated very well (*r* > 0.8) (Supplementary Table 4), thus illustrating the accuracy of our approach to measure gene expression and report differential levels of RNA species. Raw read counts were subjected to a variance-stabilization transformation as described in Anders and Huber (34) and followed by Principal Component Analysis (PCA), to partition biological and technological variability of the experiments into covariant components. Ideally, one expects the first components to capture most of the biological variability, with little non-biological effect (i.e., technical noise). On the contrary, major components poorly connected to biological variables (treatment, animals) would indicate that the dataset is dominated by noise. We found that the first component (72% of the total variance) corresponds to the species-specific variance, the second and third components (PC2, 17.8% and PC3, 9.9%) correspond to the TH treatment in one or the other species, thus showing a low level of technical noise. Since the lack of replicates did not allow us to run a proper statistical analysis, DE genes were defined as having a fold change (in log2 scale) superior or equal to +/-1, after trimmed mean of M-values (TMM) normalization using DESeq v1.14.0 (34) in blind mode and with fit-only option. Gene Ontology analysis was carried out with goProfiles 1.32.0 (35), which allows one to directly compare the GO terms associated to two DE genes lists for a direct visualization of the biological processes favored in one species vs. the other.

### RT-qPCR

Reverse transcription was carried out from 2.5 μg of total RNA, first mixed with dNTP (2 μL, 10 mM, Invitrogen) and random primers (1 μL, 50 μM, Invitrogen) in a final volume of 12 μL (with H_2_O DEPC, Ambion), and incubated at 65°C for 5 min. After hybridization, samples were put on ice prior to the addition of 1 μL H_2_O DEPC, 1 μL 0.1 M DTT (Invitrogen) and 4 μL 5X first strand buffer (Invitrogen). RNAse inhibitors (1 μL, RNAse out, Invitrogen) and reverse transcriptase (1 μL, SuperScript III, Invitrogen) were added and the reaction was incubated at 25°C for 10 min, followed by an incubation at 42°C for 40 min. Primer express (Applied Biosystems) was used to design primers (see Supplementary Table 5 for Axolotl and Supplementary Table 6 for *X. tropicalis*). Primer choice for RT-qPCR validation was optimized by combining coding sequence conservation and the reads distribution along Axolotl reference sequences. BLASTX alignments between Axolotl reference sequences and the corresponding Xenopus analogous coding sequences were visualized by dot-plot (a few illustrative examples are shown in Supplementary Figure 2). This allowed us to directly control that evidences for differential expression are unambiguously located in properly assembled transcripts, and are not an assembly artifact resulting in chimeric transcripts. qPCRs were performed on an ABI 7300 (Applied Biosystems) and analyzed with the Prism 7300 system software (Applied Biosystems). *H3f3a* and *rpl8* were selected as control genes for normalization, using Normfinder (36) (data not shown). *H3f3a* was used to compare untreated groups vs. T_3_ treated groups and *rpl8* was used to compare developmental stages. Raw data were normalized on the control gene and on the non-treated sample by the 2 CT method. Results are presented as means of Log(2CT) with standard deviation (SD). Statistical analyses, based on 3 to 11 biological replicates, were performed with the Mann and Whitney test (α = 5%).

### Network Analysis

The overlap between the sets of DE genes and KEGG pathways (Kyoto Encyclopedia of genes and Genomes database) was carried out with JEPETTO [Java Enrichment of Pathways Extended To Topology (37)], a plugin to Cytoscape v3.2.0 (38). All KEGG pathways containing at least one DE gene were collected and merged to create a global functional interaction network (see main text for description). Cytoscape was used to visualize the network and compute network properties. Hubs are defined as nodes (i.e., gene product) with a degree (a.k.a connectivity) higher than 20. Empirical cumulative degree distributions were computed with the ecdf function of the Hmisc R package. The degree distribution in *X. tropicalis* network exhibit a bimodal degree distribution (not shown). This is due to a complex of 59 DE ribosomal protein coding genes, which are highly interconnected, and as such, are characterized by a high degree. Given that they are also poorly connected to the rest of the network and form a self-contained sub-network, they were excluded from the analysis.

The identification of the dense sub-network of DE genes is based on Z-score statistics, computed by randomly shuffling the gene status (whether they are differentially expressed or not) and counting the number of DE genes in the neighborhood of actg1 and fos. This process, repeated 1,000 times, estimates the (normal) background distribution of the number of DE genes found by change. The actual number of DE genes observed around actg1 and fos is then compared to the distribution, which is used to derive the z-score (i.e., number of standard deviations from the mean) and *p*-values.

## Results

**Figure 2:**
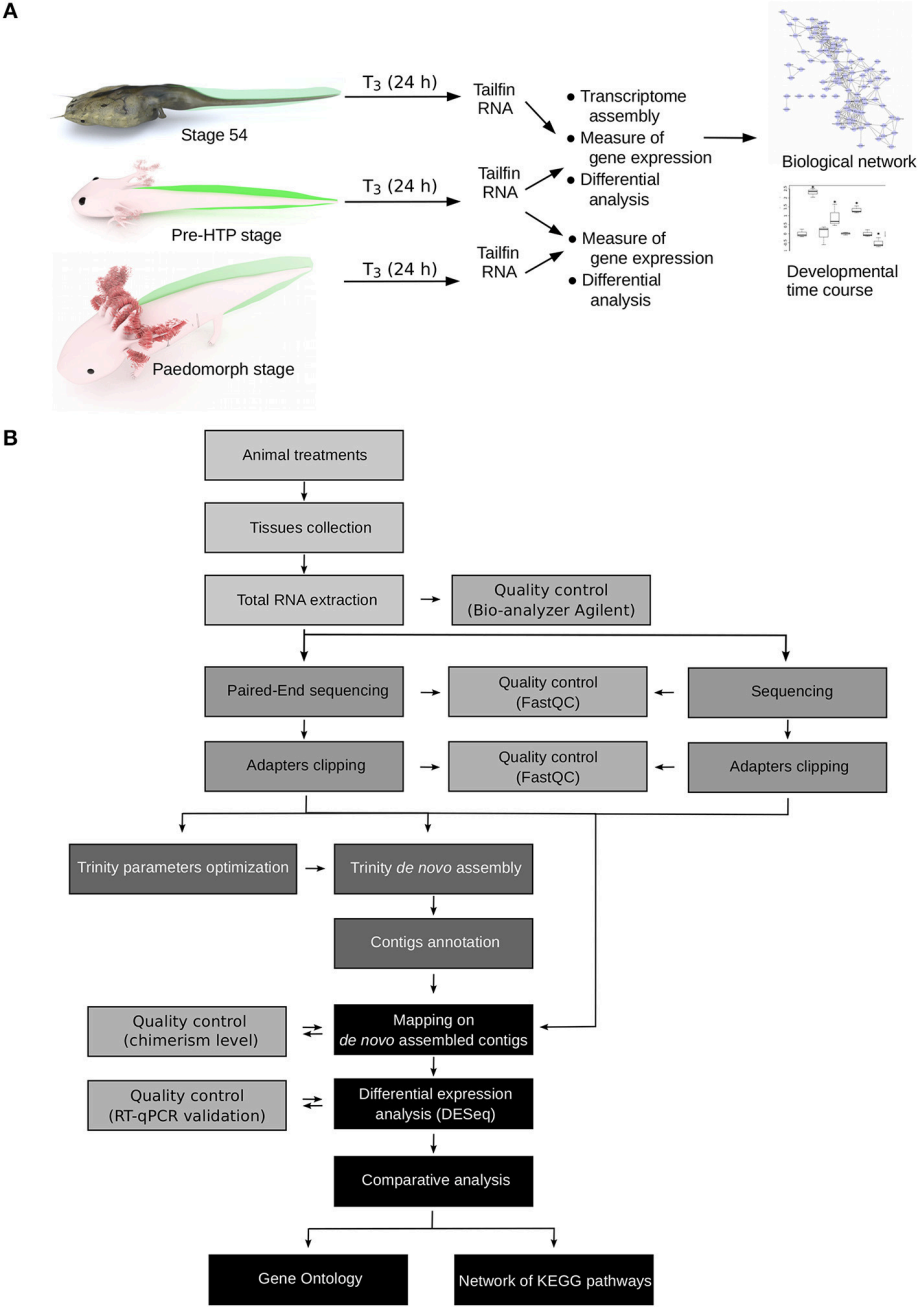
Experimental design. **(A)** Experimental setup. Axolotl and *X. tropicalis* tadpoles were treated with T_3_ for 24 h and gene expression was measured by RNA-Seq, followed by system biology analysis of gene networks and developmental profiling of gene expression. Animal paintings not to scale. Tailfin is highlighted in green. “pre-HTP” animals will refer to 2 weeks post-hatching Axolotl when endogenous TH level is low and 2 weeks before the highest level of endogenous TH (23). *X. tropicalis* tadpoles were staged according to Nieuwkoop and Faber (24). Digital paintings were carried out with BLENDER v2.8b. **(B)** Data processing workflow.

How to explain, in term of transcriptional regulation, that *X. tropicalis* and Axolotl tailfin respond differently to TH at HTP? Our experimental set up and data processing workflow to address this question are described Figure 2. Briefly, we used pre-HTP *X. tropicalis* (pre-metamorphic, stage 54) and Axolotl (Class 3 Axolotl as defined by 23), corresponding to 2 weeks post-hatching tadpoles when limb buds start growing, endogenous TH level is low and thyroid gland starts releasing T_4_. Animals were treated with 10 nM T_3_ for 24 h, tissues were collected and RNAs subjected to paired-end sequencing to measure gene expression. The choice of tissue collection at 24 h is based on the fact that in *X. tropicalis*, expression of typical DE response genes (e.g., *klf9, mmp11, thbzip*) is strongly induced as soon as 24 h post-treatment. This is also certainly true for Axolotl, where collagenase3 and stromelysin3 expression is also strongly induced of after 48 h of T_3_ treatment (21). In fact, a fast transcriptional regulation may be a general feature of T_3_ response, since it is also true in mice (39). After differential expression analysis, transcriptomic responses were further characterized by topological analysis of biological networks. We also characterized in Axolotl the transcriptional response naturally occurring at the HTP, or after T_3_ treatments at later stages (6 months old). Given that no genome sequence was available for Axolotl at the time of this work, we used RNA-Seq paired-end reads to assemble the transcriptome and generate the repertoire of coding sequences. Importantly, this also helped capture specific transcripts originating from an embryonic tissue. RNA-Seq reads were then mapped to the reference sequences to measure gene expression. In order to circumvent technological biases, and despite the availability of an improved genome sequence and annotation (40), *X. tropicalis* RNAs were subjected to the same procedure (paired-end sequencing followed by transcriptome assembly) to generate an equivalent set of reference sequences. In fact, at the evaluation step of the bioinformatic pipeline, the around 25,000 known *X. tropicalis* coding sequences were used as a gold standard to evaluate the assembly process and set optimal parameters for assembly and clustering (Supplementary Tables 1, 2). Coding sequences were then annotated by comparison to *X. tropicalis* and NCBI's nr databases. Systematic dot-plot comparison of assembled transcripts to homologous sequences together with ONT sequencing of the same RNA samples showed that the chimerism level, a typical artifact of transcriptome assembly, is low (~2%, Supplementary Figure 1) and that the vast majority of transcripts align well to their cognate homolog (see illustrative examples Supplementary Figure 2). Overall, we produced a high quality set of 17,990 *X. tropicalis* coding sequences, and 21,141 Axolotl coding sequences, that will be used as a proxy for gene reference sequences and differential analysis of gene expression. This corresponds to a set of 9,006 homologous genes common to the two species.

### An Early TH-Dependent Transcriptional Dynamic in Both Axolotl and *X. tropicalis* Tailfin

Measure of gene expression was carried out for each species by mapping the paired-end reads on the gene reference sequences that were assembled (Supplementary Table 7), and was followed by differential analysis. Principal Component Analysis (PCA, data not shown) indicates that our data set is dominated by biological signal, with very little experimental noise.

**Figure 3:**
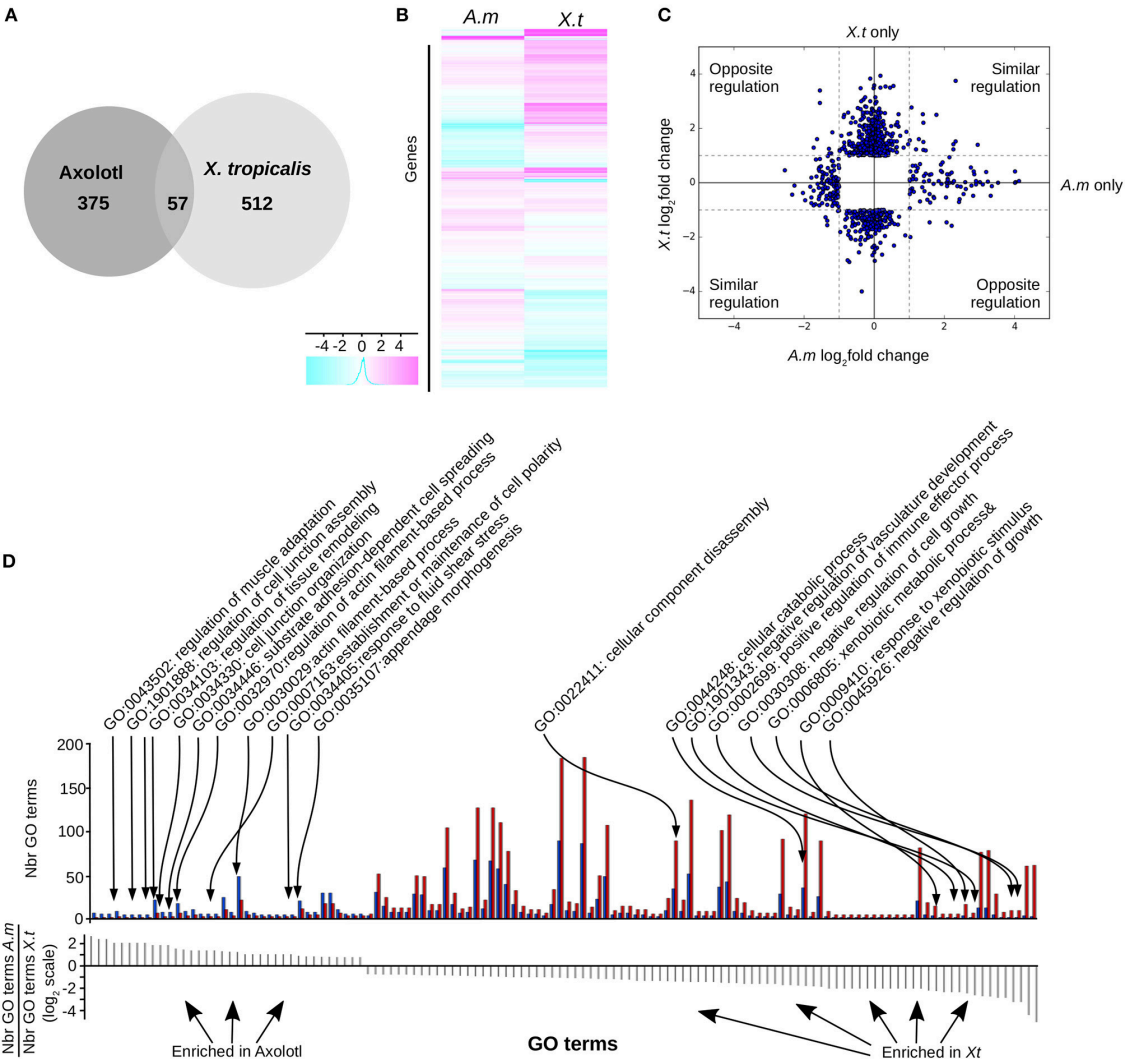
T_3_ regulates different gene sets in Axolotl and *X. tropicalis* at the post-embryonic transition. **(A)** Overlap between differentially expressed genes in both species. **(B)** Heatmap of differentially expressed genes in Axolotl (A.m) and *X. tropicalis* (X.t). **(C)** Log2 ratio of TH-induced gene expression changes. **(D)** Gene ontology analysis. Top: Number of genes for each GO term (not shown), on both species. Blue: Number (Nbr) of terms found in Axolotl. Red: Number of terms found in *X. tropicalis*. Bottom: ratio of the number of terms found in both species for each GO term (in the same order as the top panel). Positive and negative values correspond to terms mostly found in Axolotl or *X. tropicalis* gene set, respectively.

We found (Figure 3) 569 (6.3%) differentially expressed (DE) genes in *X. tropicalis*, 303 up- and 266 down-regulated, and 432 (4.8%) in Axolotl, 153 up- and 279 down-regulated (see Supplementary Tables 8, 9, respectively). These genes include known TH responsive genes previously described for both species, such as *klf9* and *mmp11* (21). A number of genes belonging to the TH signaling pathway (*thra, dio2*, and *thbzip*) have been filtered out because of low reads count, thus making any differential measure of their expression hazardous. For *thrb*, this agrees well with the work of Safi et al. (21), who also reported a very weak expression level in Axolotl. For Axolotl, we confirmed the differential expression status of 20 up and down regulated genes by RT-qPCR (Figures 5C, 6, Supplementary Figure 3). For *X. tropicalis*, the DE gene list is well in line with previously published micro-array experiments (41), and has been validated experimentally (Supplementary Figure 4), together with seven additional genes by RT-qPCR (Figure 5D). These results unambiguously show that Axolotl tailfin responds to T_3_ at the transcriptional level, to an extend somewhat similar to *X. tropicalis*. Only a few genes (57 genes) were T_3_ responsive in both species (Figure 3, Supplementary Table 10), of which 32 exhibited a common regulation (14 up- and 18 down-regulated). This subset of co-regulated genes includes the well-known TH responsive gene *dio3* and is also enriched in transcription factors such as *brca1, fosl2, klf13, klf17, klf9, nr4a2, sox4*, and *znf395*. Except for actg1 and fos, genes with opposite regulation in the two species *(rhof, ankrd1, lamc2, epb41l3, itga11, hhipl2, crispld2, cdh2*…, Figure 3C) are exclusively composed of membrane bound or extracellular matrix proteins.

We next carried out Gene Ontology analysis in order to contrast the “biological processes” GO terms found in the gene sets of both species. This analysis revealed significant alternative usage of GO terms (Fischer test, *p* < 10^−4^, see Materials and Methods) between species (Figure 3D, Supplementary Table 11). In Axolotl, DE genes are involved in various aspects of tissue remodeling (e.g., cell junctions organization, cell adhesion, extracellular matrix (ECM) organization and structure) together with a number of terms related to actin biology. In contrast, in *X. tropicalis*, DE genes are more involved in diverse catabolic processes, disassembly of cellular components, regulation of cell growth, and several components of the immune system, fitting well the known biology of tailfin resorption programs.

These results show that as soon as 2 weeks post-hatching, T_3_ treatment induces the differential regulation of many genes in Axolotl tailfin, including genes often associated with TH transcriptional responses. Therefore, and this is an important result, the HTP corresponds to a TH sensitive period in Axolotl tailfin, despite the lack of visible anatomical change. The programs induced in Axolotl and *X. tropicalis* differ significantly, except for a core set of genes involved in TH signaling and transcriptional regulation. Likewise, transcriptional responses contrast sharply in term of biological processes (remodeling of the acto-myosin network vs. tail resorption program).

### Integrated Functional Relationships Between DE Genes

**Figure 4:**
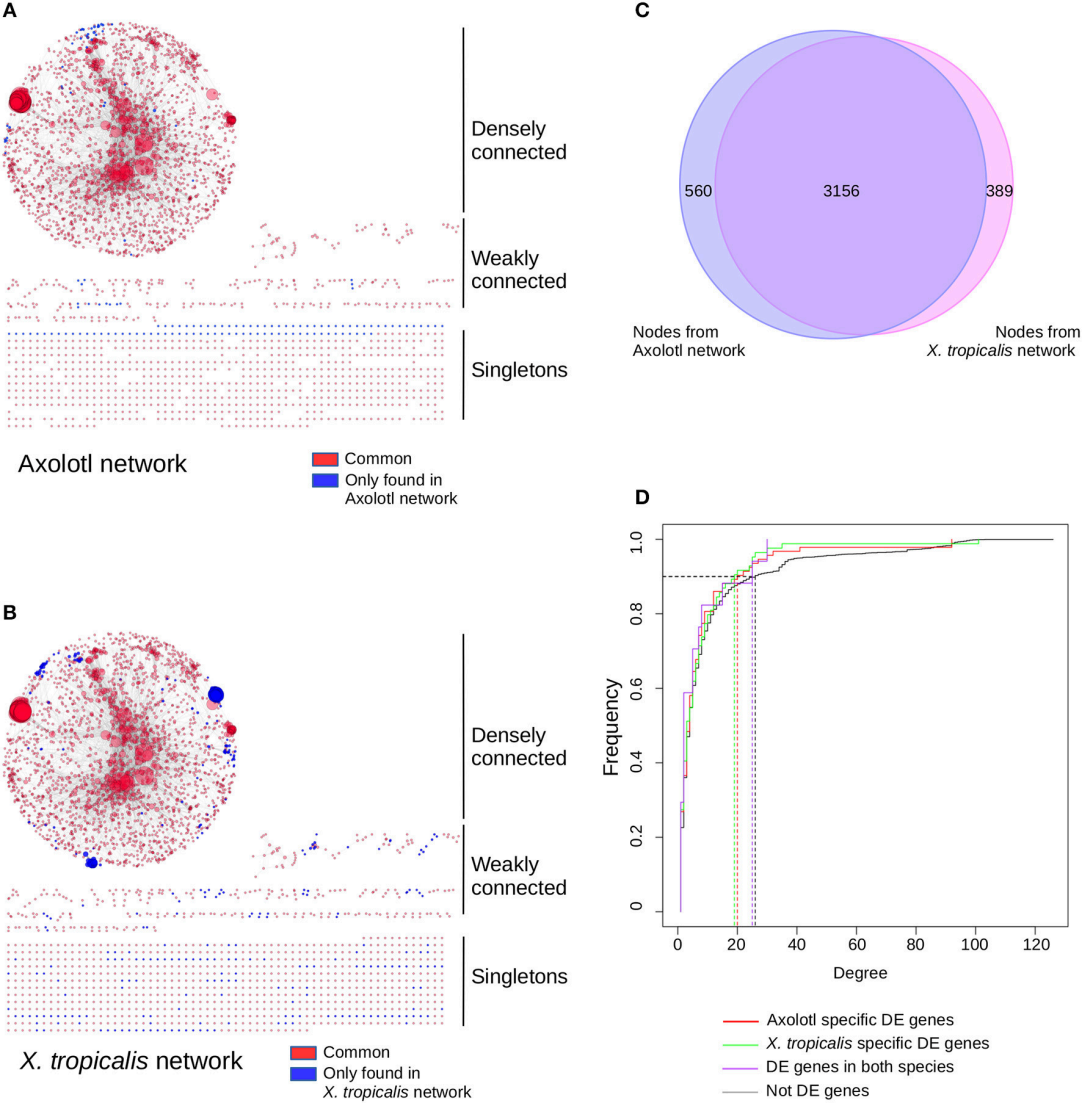
T_3_ affect a similar network of pathways in both species, despite regulating different sets of genes. Networks of KEGG pathways affected in Axolotl **(A)** and *X. tropicalis* **(B)**. The reconstructed network for Axolotl is composed of 3,305 nodes and 12,776 edges, with a densely connected component (2,274 nodes and 12,554 edges), some weakly connected genes (273 genes and 222 edges) and a set of singletons (758 nodes). The *X. tropicalis* network is composed of 3,561 nodes and 16,237 edges, with a highly interconnected component (2,443 nodes and 15,950 edges), some weakly connected genes (318 genes and 287 edges) and a set of singletons (800 nodes). Nodes correspond to gene products, linked together by the functional interactions described in the pathways (edges). Individual node size is proportional to the number of nodes connected to it. Large nodes thus correspond to hubs between KEGG pathways. Red: nodes in common to both networks. Blue: nodes only found in one (or the other) network. Layout computed with the prefuse force directed algorithm. **(C)** Overlap between the node (gene product) content of the two networks. **(D)** Cumulative distribution of node connectivity (degree). In both species, T_3_ do not target (or avoid) specific network components.

To get a functional and integrated view of the molecular phenotype described by our dataset, we took advantage of the KEGG pathways (42), a collection of curated functional interactions organized in a number of well-identified pathways. Given that components are often shared between pathways, we undertook to reconstruct a network of all the KEGG pathways having at least one DE gene (i.e., not all genes are DE in the network). In this network, nodes represent gene products and edges represent functional connections (phosphorylates, activates, represses…) between them. The first advantage of this approach is to provide an integrated view of the functional interactions between gene products and thus address how T_3_ response is orchestrated between biological pathways, and whether this response is different between the two species. In Axolotl, 116 genes (out of 412 DE genes) could be assigned to 112 KEGG pathways (Supplementary Table 12). In accordance with our GO enrichment analysis (and despite the medical orientation of their name), the most represented pathways have strong components related to actin, cytoskeleton and ECM biology: “ECM-receptor interaction,” “Hypertrophic cardiomyopathy (HCM),” “Dilated cardiomyopathy,” “Focal adhesion.” In *X. tropicalis*, 171 (out of 569 DE genes) could be assigned to 136 KEGG pathways (Supplementary Table 13), with many metabolism-related pathways. The reconstructed networks are shown Figure 4. The Axolotl network has a total of 3,305 nodes and 12,776 edges, together with 758 singletons (unconnected nodes). The *X. tropicalis* network is composed of 3,561 nodes and 16,237 edges, with 800 singletons. From the 57 T_3_ responsive genes common to both species, 19 mapped to KEGG pathways and 14 were located in the highly connected components of the two networks. Remarkably, the two networks were highly similar with 99 KEGG pathways and 3,156 nodes in common (95% of nodes from Axolotl network and 89% of *X. tropicalis*, Figure 4C), illustrating that despite a limited overlap between DE genes sets, T_3_ response affects similar pathways in both species.

The second advantage of a network of pathways is to identify integration points between multiple pathways within a network (i.e., gene products shared between multiple pathways), which ensure communication and signal propagation between subnetworks. They are well-known to have a strong structural role in biological networks, which is (in part) responsible for their robustness, and functionally targeting them has a strong predicted biological impact (43). This is the underlying postulate of our approach: regulating the expression of a gene product shared between multiple pathways will likely have large biological effects since this will simultaneously affect multiple pathways. Here, we addressed how T_3_ response preferentially targets (or avoids) the gene products shared between multiple pathways and how this differs between Axolotl and *X. tropicalis* responses. More formally, gene products shared between pathways correspond to highly connected nodes (“hubs”) and the question can simply be put as the relationship between biological responses (DE/non- DE) and node connectivity. We first characterized the relationship between the degree (level of connectivity) of DE genes and T_3_ response, in both species, in order to characterize the transcriptional dynamic of their network. The connectivity of a node is measured by a simple metric, the degree, which corresponds to the total number of edges (i.e., functional interactions) connected to it. We thus plotted the cumulative degree distribution of DE genes and non-DE genes. This analysis is akin to ROC curve analysis. We found a similar distribution for Axolotl and *X. tropicalis* DE genes, as well as non-DE genes (Figure 4D). As a result, in one species vs. the other, the TH response (1) affects nodes with similar connectivity and (2) does not target a specific subset of high or low connectivity. These results clearly show that although the DE gene sets are different in both species, those described in KEGG pathways belong to (almost) identical networks and they have similar transcriptional impact on the network as a whole.

**Figure 5:**
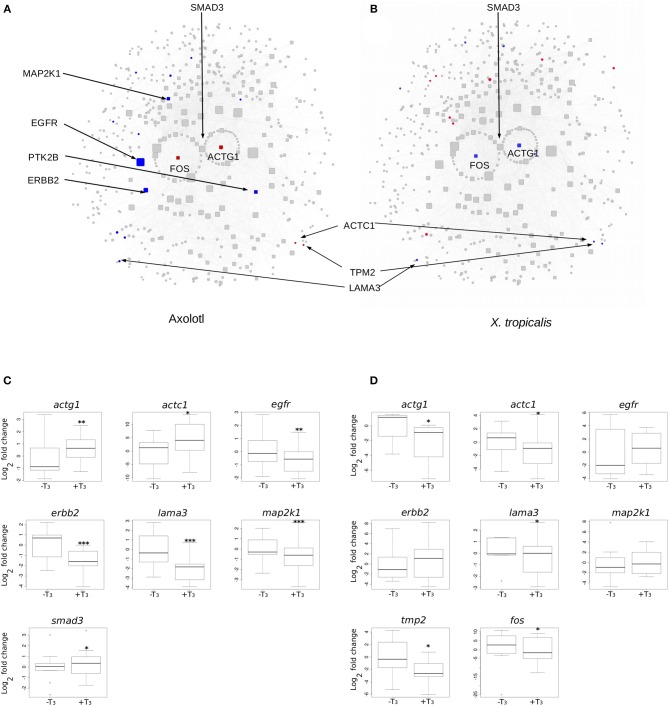
Differential gene expression at the Actg1-Fos subnetwork, in Axolotl and *X. tropicalis*. **(A)** Axolotl subnetwork. **(B)** *X. tropicalis* subnetwork. The subnetworks are composed of the first (laid out in circle) and second neighbors of Actg1 and Fos nodes. Hubs (nodes with degree >20) are shown with rounded squares. Node size is proportional to their degree (connectivity). Colors indicate differentially expressed genes (red: induced, blue: repressed). Homologous nodes are located at the same place in both networks. **(C)** RT-qPCR analysis of DE genes in the Axolotl subnetwork. **(D)** RT-qPCR analysis of DE genes in the *X. tropicalis* subnetwork. Statistical significance (Mann-Whitney test) with ^*^p ≤ 0.05, ^**^p ≤ 0.01, ^***^p ≤ 0.001.

We next focused on hubs (highly connected nodes). Overall, Axolotl and *X. tropicalis* networks contain 309 and 422 hubs, respectively, among which 66 correspond to DE genes in one or the other species. There is a total of five DE hubs specific to Axolotl (*aqr, ptk2b, erbb2, map2k1, egfr*), all down-regulated (Table 1). Remarkably, four of them are involved in Pi3k/Akt signaling, suggesting that T_3_ treatment strongly impacts this second messenger signal transduction pathway. A total of 57 hubs are only differentially expressed in *X. tropicalis*, corresponding mainly to ribosomal protein coding genes, together with *mcm5, psme3, polr2h, lsm2, fau*, and *irs2* (listed in Table 1, without 17 the ribosomal protein coding genes) which are involved in metabolism, regulation of gene expression, and DNA repair. Strikingly, only two hubs correspond to DE genes in both species (*actg1* and *fos*), although with opposite regulation (both genes up-regulated in Axolotl and down-regulated in *X. tropicalis*) (Table 1). These two nodes are very close in the networks and are separated by a single node, *smad3* (Figures 5A,B respectively, for Axolotl and Xenopus), for which the differential expression level in Axolotl is well-below our threshold. However, extensive RT-qPCR analysis with a high number of biological replicates confirmed its DE status (Figures 5C, 7). Apart from Smad3, Fos and Actg1 are not directly connected to other DE genes (first neighbors) but their second neighbors are. In fact, these second neighbors are particularly enriched in DE genes (19 for Axolotl, Figure 5A, and 13 for *X. tropicalis*, Figure 5B). In Axolotl, these DE genes are mainly down-regulated (16/19) and are involved in cell migration and cell apoptosis. In *X. tropicalis*, they do not show any preferred direction of change. They are mainly involved in cell proliferation, cell differentiation, cell survival, and oxidative stress. Three of these DE genes are common between the two species (*actc1, tpm2*, and *lama3*). The first two have opposite regulation, where mRNA levels increase in Axolotl and decrease in *X. tropicalis*. In both species, *lama3* mRNA level decreases following T_3_ treatment (Figure 5). Interestingly, this subnetwork clusters most (four out of five; all except *aqr*) of the hubs that are differentially expressed only in Axolotl (Figure 5A). This represents an unusually high concentration of DE hubs around *actg1* and *fos* (*z*-score = 2.029, *p* = 0.0424). The fact that this subnetwork exists in alternative transcriptional states in both species at HTP is an important result and is noteworthy. The species-specific regulation of the components of this subnetwork has been confirmed by RT-qPCR for both Axolotl and *X. tropicalis* (Figures 5C,D, respectively). Overall, these results show that although the transcriptional response to TH mobilizes similar networks of biological pathways in both species, their transcriptional changes display sharp contrasts: (1) T_3_ affects different network components in both species, and (2) in (and only in) Axolotl, the Fos-Actg1 subnetwork contains most of the hubs that are differentially expressed.

**Table 1:** List of hubs (genes) differentially expressed in either Axolotl or *X. tropicalis*.

**Gene name**	**Axolotl_log2FC**	***X. tropicalis*_log2FC**	**Degree**	**Neighborhood connectivity**
**AXOLOTL SPECIFIC HUBS**				
AQR	−1.67	0.9	92	85.59
EGFR	−1.77	0.26	92	24.63
ERBB2	−1.71	0.3	41	32.24
PTK2B	−1.33	−0.25	27	43.96
MAP2K1	−1.02	−0.18	24	31.58
**HUBS REGULATED IN BOTH SPECIES**				
ACTG1	1.42	−1.96	30	12.6
FOS	1.39	−2.26	25	27.08
***X. tropicalis*** **SPECIFIC HUBS (EXCLUDING RIBOSOMAL PROTEINS)**				
LSM2	0.1	1.25	101	79.47
FAU	0.19	−1.59	72	67.29
PSME3	0.08	1.58	35	34.23
POLR2H	0.21	1.16	26	14.08
IRS2	−0.57	1.13	25	34.04
MCM5	−0.32	−1.44	24	22.67

*FC, Fold change*.

### Transcriptional Response to T_3_-Treatment Correlates With Development-Dependent Changes of Gene Expression

**Figure 6:**
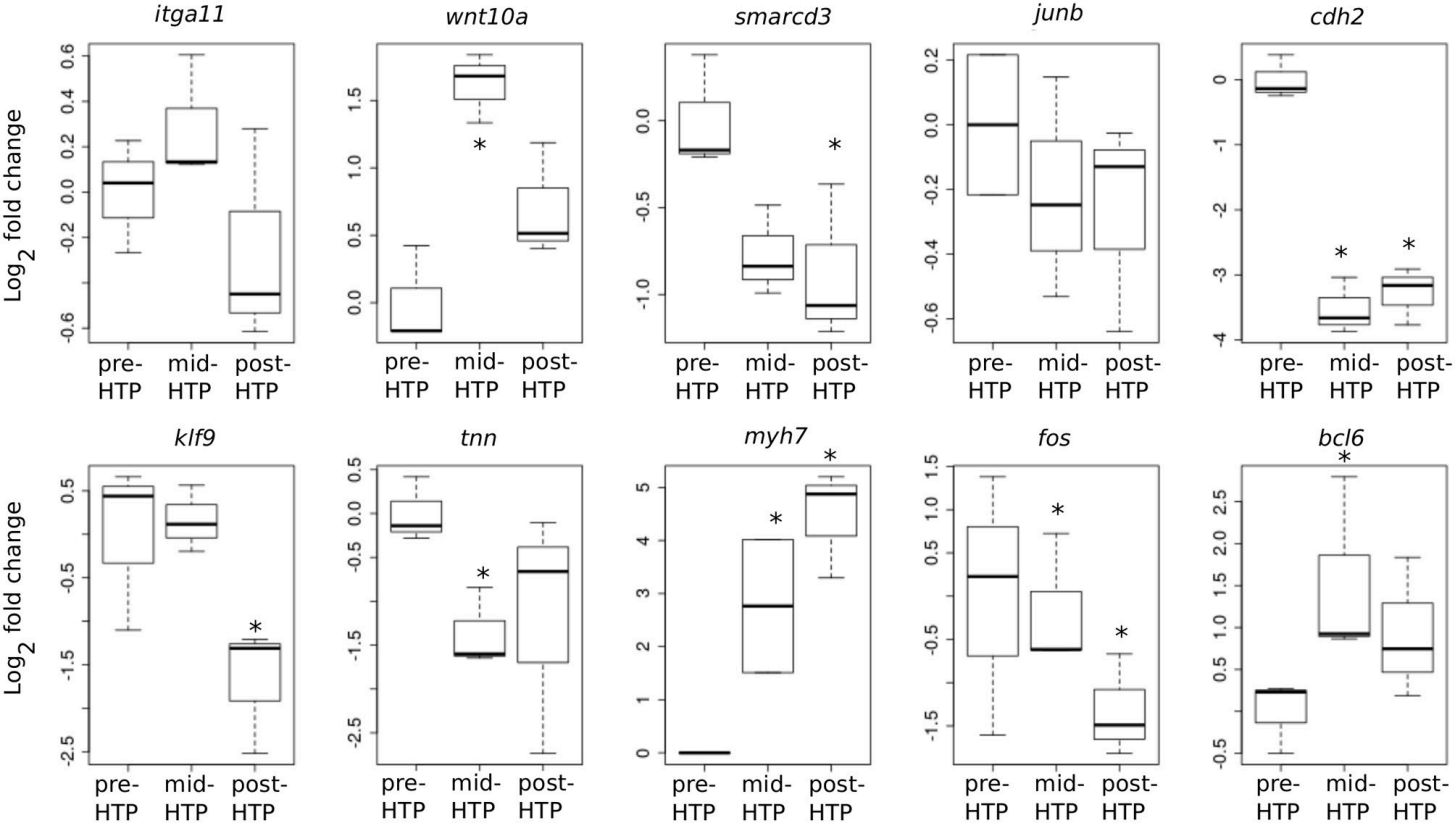
Developmental time course of gene expression. Normalized gene expression changes (log2 Fold Change) before, during or after the endogenous peak of TH, corresponding to pre-, mid-, and post-HTP animals. Statistical significance (Mann-Whitney test) with ^*^p ≤ 0.05.

We next addressed whether T_3_-induced gene expression changes recapitulate gene expression changes during normal development in Axolotl. To this end, we carried out quantitative RT-PCR on tailfin mRNA samples extracted from pre-HTP (2 weeks post-hatching when limb buds starts growing, refer to class 3 Axolotl as defined by 21, Figure 1), mid-HTP [1 month post-hatching, i.e., class 8 animals as defined by Rosenkilde et al. (23), corresponding to the maximum of the peak, Figure 1] and post-HTP animals (3 months old animals, refer to class 12 Axolotl as defined by 21, where TH endogenous level dropped significantly, Figure 1). Of the 10 genes tested, five displayed similar transcriptional responses after T_3_ treatment and during the course of development (*bcl6, cdh2, myh7, smarcd3, tnn*, Figure 6). *Junb, klf9* and *itga11* are not DE between the two developmental stages, while *junb* and *klf9* were up-regulated and *itga11* down-regulated following T_3_ treatment at pre-HTP. Only, *wnt10a* showed an opposite transcriptional response to T_3_ vs. developmental changes. We also note that the mRNA levels for *klf9*, a typical T_3_-induced gene in tetrapods, decreased during later stages of development (Figure 6) as previously shown for *X. tropicalis* (44). This shows that T_3_ treatment at pre-HTP induces changes of gene expression that mostly parallel endogenous transcriptional profiles changes through this transition. This also further confirms that Axolotl tailfin responds to endogenous TH as early as 2 weeks post-hatching, despite no apparent anatomical change.

## A Distinct T_3_ Transcriptional Responses at HTP and Paedomorph Stages

**Figure 7:**
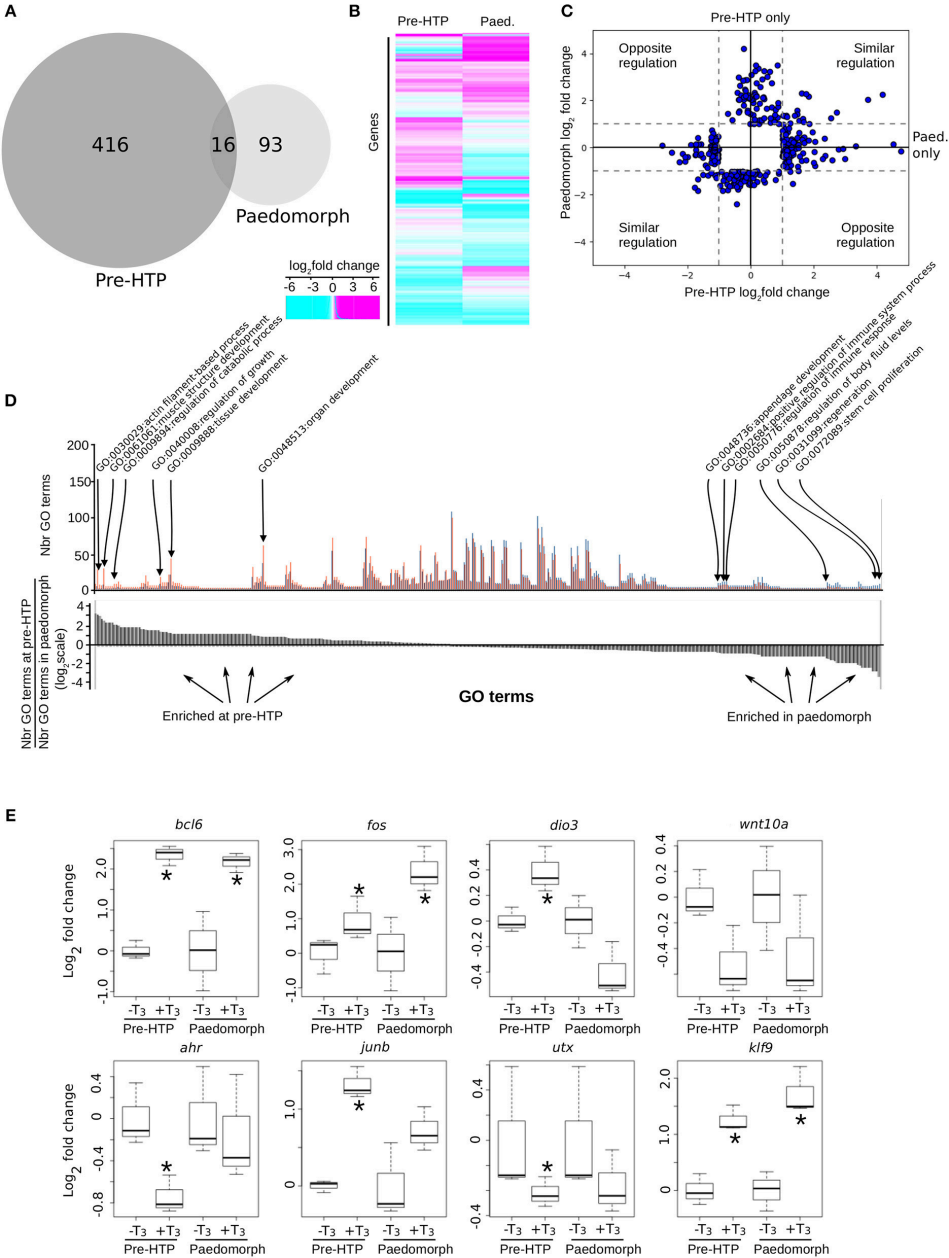
Axolotl tailfin transcriptional responses to T_3_ at pre-HTP and paedomorph stages. **(A)** Overlap between T_3_-responsive gene sets at pre-HTP and 6 months old paedomorph stages, measured by RNA-Seq. **(B)** Heatmap of differentially expressed genes at pre-HTP and paedomorph stages. **(C)** Expression fold changes at pre-HTP vs. old paedomorphs. **(D)** Gene ontology analysis. Top: Number of genes for each GO term (not shown), at both stages. Red: Number (Nbr) of terms found at pre-HTP. Blue: Number of terms found in paedomorph. Bottom: ratio of the number of terms found at each stage for each GO term (in the same order as the top panel). Positive and negative values correspond to terms mostly found at pre-HTP or paedomorph gene set, respectively. **(E)** RT-qPCR normalized gene expression changes (log2 Fold Change) after T_3_ treatment. Tailfin transcriptional response to T_3_ differs between class 3 larvae and 6 months old paedomorphs. Statistical significance (Mann-Whitney test) with ^*^p ≤ 0.05.

We next addressed whether the ability of Axolotl tailfin to respond to TH changes through development. To this end, we treated pre-HTP tadpoles and adult peadomorphs (6 months old post-hatching) with T_3_ for 24 h and measured gene expression changes by RNA-Seq (see Materials and Methods, Figure 7). In paedomorphs, we found 109 genes differentially expressed upon T_3_ treatment (Figures 7A–C, Supplementary Table 14), from which only a minority (16/109) was in common with the pre-HTP TH-responsive genes set (*bcl6, klf9, cdh2, chst6, dnajb5, fos, hspb1, klf17, mcoln1, frkb, pla2g7, pprc1, prdm1, sox4, tmprss4, ulk4*). Importantly, none of these DE genes (except *fos*) belong to the *Actg1-Fos* subnetwork, which strongly suggest that the this subnetwork is in an alternative transcriptional state at this stage. This view is also supported by the fact that *actg1* expression is not TH responsive in the paedomorph. Altogether, these results highlights the sharp contrast between TH responses at pre-HTP and paedomroph stages. Gene ontology analysis further confirms this result and shows that biological processes are differentially affected at pre-HTP and paedomorph stages (Fischer test, *p* = 0.027900, see Materials and Methods). At pre-HTP, TH response impacts several developmental processes and actin/muscle biology, which contrasts with paedomorph where TH response focus more on immune system, various differentiation processes and regeneration (Figure 7D, Supplementary Table 15).

We also carried out additional validation with a set of previously identified T_3_ responsive genes, by RT-qPCR, and found good agreement with RNA-Seq data (Figure 7E). Interestingly, some genes (*bcl6, fos*, and *klf9*) displayed a similar response between the two stages, whereas for other (*ahr, junb, utx*), T_3_ responsiveness reached significance only at the pre-HTP stage, despite a weak, but similar, trend (Figure 7C). Of note, *wnt10a* almost reached statistical significance, but failed because of the large biological variability of the samples. The three genes that are members of a core set of genes idiosyncratic of a TH response (7), transcription factors *bcl6, fos*, and *klf9*, display a similar response at 2 weeks post-hatching and in adult. These results strongly suggest that despite its TH responsiveness, tailfin engage specific and distinct responses at different stages. This further strengthens the fact that Axolotl tailfin at HTP undergoes a very specific TH-dependent transcriptional program.

## Discussion

TH mediate diverse transcriptional responses in a cell- and/or tissue-specific manner. To dissect the molecular basis underlying the variety of these biological responses, we used the well-known and evolutionary conserved post-embryonic development (frog metamorphosis, perinatal period in mammals…) as a model of TH response (9). Interestingly, Axolotl and *X. tropicalis* tailfins are homologous tissues, but yet respond differently to T_3_ at a similar developmental period (limb development at HTP). This transition, quite extreme in anuran amphibians, corresponds to the abrupt and profound change of body shape known as metamorphosis. In contrast, Axolotl shows little or no sign of post-hatching transition, despite a transient surge of TH (23). Since these early works, the apparent lack of transition at this stage in Axolotl has been interpreted as a lack of TH action by some unknown mechanism (45, 46). This point has been questioned later because at this stage, thyroid signaling is already fully operational and T_3_ treatment results in accelerated growth of larval features (22). In order to resolve the molecular determinism of this apparent contradiction, we set out to compare the tailfin transcriptional response to T_3_ in both species. In this work, we first show that Axolotl tailfin strongly responds to T_3_ at a period of high levels of endogenous TH (around 2 weeks post-hatching), despite no visible anatomic change. T_3_ responsive gene sets are different between Axolotl and *X. tropicalis* but belong to the same pathways, and mirror phenotypic differences (tailfin resorption vs. maintenance). In term of regulatory mechanism of TH action, we next sought for a possible molecular subnetwork that may act as a switch that controls tailfin developmental fate. By coupling functional genomics to network biology, we could identify an Actg1-Fos subnetwork switching to alternative transcriptional states in both species, and that parallels tailfin fate.

### Thyroid Hormones Induce Alternative Molecular Phenotypes in Axolotl and *X. tropicalis*

Despite limited overlap between DE gene sets, a few genes idiosyncratic of TH response (*mmp11*, fos, *klf9*) (7, 47, 48) are also differentially expressed in the 2 weeks post-hatching Axolotl. This important result not only shows that tailfin responds to T_3_, but also that the transcriptional program is different from the resorption program induced in *X. tropicalis* tailfin. Tailfin responsiveness to T_3_ is further supported by (1) change in gene expression *in vivo* that parallels endogenous variations of TH levels [this work and Rosenkilde et al. (23)], and (2) the fact that despite different DE gene sets (see below), the same molecular pathways are mobilized in both species. In fact, the Axolotl tailfin transcriptional response is not limited to this small subset of genes but includes 432 genes, affecting various cellular processes. While a catabolic response predominates in *X. tropicalis*, Axolotl tailfin seems to respond mainly by engaging a developmental process. In particular, there is a strong enrichment in terms related to reorganization of the cytoskeleton and the acto-myosin network, suggestive of a transition or a remodeling of the tailfin. This is interesting because this would imply that the tailfin of young (i.e., 2 weeks post-hatching) and older (paedomorph) animals are not equivalent. In term of Axolotl biology, this raises the interesting possibility that paedomorph tailfin may have different mechanical and physical properties, maybe more suited to the increased body size of a fully-grown animal with long-term aquatic life style. This would then make the case for an early TH-sensitive period in *Xenopus* and Axolotl, that would initiate tailfin resorption or remodeling, respectively.

Strikingly, and perhaps not surprisingly, the transcriptional response in the two species was very different, with only 32 genes exhibiting similar regulation. Interestingly, 8 of these 32 genes are known transcription factors that may regulate the expression of large subsets of genes depending on the species-specific and tissue-specific chromatin context or co-regulators expression. A total of 25 genes also show opposite regulation, mainly linked to actin-myosin networks and metabolism, supportive of very different cell fates and organ outcomes. It is noteworthy that the only transcription factor in this list is Fos (see below). These different transcriptional responses (and the resulting phenotypes) in the two species could also reflect differences in the sensitivity or competency of the cells/tissues to respond to the T_3_ stimulus. The regulation of THR expression or genes involved in TH availability is quite different between the two species. As expected, we confirmed that the two types of THR (α and β) are weakly expressed and not differentially regulated following T_3_ treatment in Axolotl larvae (21). In *X. tropicalis* tadpoles, *thrb* mRNA levels are strongly induced following T_3_ treatment; a hallmark of metamorphosis. In contrast, D3 deiodinase, the key enzyme involved in the degradation of biologically active hormone at target tissues (49), shows little species-specific transcriptional responses and its expression is induced in both Axolotl and *X. tropicalis*, following TH treatment (respectively, 4.8 vs. 2.4 fold). Interestingly, older Axolotl animals lose the ability to regulate *dio3* mRNA level in a T_3_-dependent manner.

The different transcriptional responses could be mediated by the species-specific differential T_3_-regulation of pioneer factors and/or genes involved in histone modification or DNA-methylation. Pioneer factors are proteins contributing to cell type-specific transcriptional competence by binding to and decondensing chromatin (50). Indeed, we observed different T_3_ effect for several forkhead box transcription factors, such as Foxp4, Foxa2, and Foxp1 (down-regulated in Axolotl) or Foxo1 (up-regulated in *X. tropicalis*). In addition, the chromatin state landscape could also be quite different between the two species, as suggested by the large set of T_3_ regulated chromatin modifying factors: Kdm6b (up), Men1 and Phf8 (down) in Axolotl, Carm1, Dot1L, Ezh2, Kdm6b, and Smarca4 (up) in *X. tropicalis*. The role of DNA methylation and histone methylation in metamorphic processes is currently being actively studied. For example, metamorphosis in lampreys has been associated with DNA methylation (51), and T_3_-induced histone modifications have been shown to be part of the mechanism of THR action during amphibian metamorphosis (3).

### Actg1 and Fos, Two Hubs With Opposite Regulation in Both Species

As described above, the Axolotl transcriptional response to T_3_ is vastly different from that found in *X. tropicalis*. This certainly reflects the opposite tailfin fate (maintenance vs. resorption) characteristic of both species. We identified a small number of biologically relevant genes of interest, through a straightforward network analysis. To this end, we used KEGG pathways as a set of high-quality resources that aggregate a curated knowledge of functional interactions between gene products, organized in independent pathways devoted to specific topics. By building a network of KEGG pathways components, we reconstructed a network of functional interactions which provides us with (1) an integrated view of the global impact of T_3_ in the two species, and (2) a data type suitable for formal exploration with the rich framework of network analysis. Our analysis of node topology quickly identified hubs which, by definition, are nodes (gene products) shared by several biological pathways. Affecting their biological activity is likely to have large biological consequences and as such, they are often considered as weakness points in biological networks (43). Remarkably, the two networks (one build for each species) are almost identical despite the very poor overlap between the DE gene lists of *X. tropicalis* and Axolotl, since they both use overlapping sets of KEGG pathways. This nicely illustrates the connections between network evolution, adaptation and biological robustness (52): given a similar network topology, dynamic changes of networks states are flexible enough to accommodate different life history traits. In terms of transcriptional changes in the biological networks, our analysis provides a number of interesting observations, which may help understand the molecular switch that generates alternative functional output and how they translate into opposite organ fates.

The key point is that the Actg1-Fos subnetwork (i.e., a collection of hubs clustered around Fos and Actg1) displays alternative transcriptional states between Axolotl and *X. tropicalis* at pre-HTP, and between pre-HTP and paedomorph stages. By definition, all the components of this subnetwork are functionally connected and collectively contribute to a number of biological pathways, ultimately translating into a coordinated biological process (e.g., tailfin regression or maintenance). We proposed that this sub-network is one of the molecular determinants of the differential response to T_3_ in both species and at both stages in Axolotl. In this context, two hubs are remarkable: *fos* and *actg1*, because they are differentially expressed in both species, but display opposite regulation. The first hub is Fos, which is induced in Axolotl and repressed in *X. tropicalis*. This is a transcription factor that regulates a large subset of genes involved in cell proliferation, differentiation and apoptosis (53). In fact, a recent survey showed that it belongs to a gene set idiosyncratic of TH responses (7). Its ability to control such a large collection of biological processes stems from its mode of action. Fos is a subunit of AP1, which forms homo- or hetero-dimers with other basic region-leucine zipper proteins that all belong to the subfamily of Jun, Fos, Maf, and Atf transcription factors. The opposite effects of AP1 on cell death and survival results from the transcriptional activation of a combination of positive and negative regulators of apoptosis. Interestingly, in Axolotl, *fos* is T_3_-responsive at both larval and adult stages whereas *junb* and the other components of the Actg1-Fos subnetwork are not. This leads to the attractive possibility that tailfin fate is governed by the relative ratio of AP1 components regulated in a T_3_-dependent manner. In addition, the fact that *fos, junb* and the Actg1-Fos subnetwork display different expression responses between *X. tropicalis* and Axolotl suggests alternative cell fate commitments. Interestingly, the functional connection between Fos and THR is not new, since they are known to share many target genes, on which they act reciprocally to repress the transcription induced by the other (54–56).

The second hub highlighted by our analysis corresponds to *actg1*, which is induced in Axolotl and repressed in *X. tropicalis*. Actg1 is a central element of the acto-myosin network. It is noteworthy that *actg1* null mice are viable during embryonic development, but most die shortly after birth at the post-embryonic transition (57). Actin plays also a key role in apoptosis (58), although its precise mechanism remains elusive. Our network analysis that links functionally Actg1 and Fos by a single node implies that Actg1 may have a nuclear localization. This is now clearly established, and Actg1 is known to participate with transcriptional gene activation (for all three RNA polymerases), editing and nuclear export of mRNAs, DNA repair, chromatin remodeling, development and transcriptional reprogramming (59, 60). In addition, Actg1 and Fos are known to belong to the same synexpression group, since their transcription is controlled by a similar serum response element in their promoter (61).

Gene regulatory networks have a modular structure, with sub-circuits dedicated to specific tasks (62) which can be recruited multiple times in different contexts during the course of evolution (63). The evolution of a regulatory module's output can easily account for the differential recruitment of the Fos-Actg1 subnetwork. Obviously, the step forward would be to address the mechanistic details and decrypt their transcriptional regulatory mechanisms. Unfortunately, our ChIA-PET analysis in *X. tropicalis* shows that neither *fos, actg1* nor the other components of the Actg1-Fos subnetwork belong to the repertoire of genes whose expression is directly controlled by the TH receptor (39 and data not shown), and the transcription factors involved remain to be determined. Thus, even if *fos* and *actg1* are direct TH target genes in Axolotl (which we currently do not know), the comparison between the two species is not trivial.

In this work, we show that despite the lack of visible anatomical changes, Axolotl tailfin responds to known endogenous variations of T_3_ levels, at the transition between tadpole and paedomorph stages (2 weeks post-hatching). Compared to the transcriptional response to T_3_-treatment at similar developmental period in *X. tropicalis* (metamorphosis), TH signaling acts on different target genes, as illustrated by vastly different DE gene sets. However, the two transcriptional response are mediated by remarkably similar cellular pathways in two species with opposite tailfin fate (maintenance vs. resorption). We also show that tailfin fate correlates with the alternative transcriptional state of a dense subnetwork around fos and actg1. We propose that the transcriptional state of this subnetwork help explains why two similar tissues and at a similar developmental period, respond differently to TH at HTP.

## Data Availability

The datasets generated for this study can be found in SRA, under the references SRP067617, SRR810197, SRR8101977 and PRJNA498010.

## Author Contributions

NB and LMS designed the experiment. GK, NB, and MR carried out the experiments. GK, LMS, and NB processed and analyzed the data. CB and CF sequenced RNA samples. GK, NB, and LMS wrote the manuscript.

### Conflict of Interest Statement

The authors declare that the research was conducted in the absence of any commercial or financial relationships that could be construed as a potential conflict of interest.
