# Supplementary material for: Opposite T3 Response of ACTG1–FOS Subnetwork Differentiate Tailfin Fate in Xenopus Tadpole and Post-hatching Axolotl
Source: Front Endocrinol (Lausanne). 2019 Apr 2;10:194. doi: 10.3389/fendo.2019.00194 (PMC6454024; doi:10.3389/fendo.2019.00194)
Supplement: Supplementary file 1 [file Data_Sheet_1.PDF]

Supplementary Material

1 Supplementary Data

Illumina reads:

Axolotl raw data have been deposited on the Short Read Archive under accession numbers SRP067617, SRR810197 and SRR8101977, and X. tropicalis under accession numbers PRJNA240154.

Oxford Nanopore Technology reads:

Axolotl aw data deposited under the SRA reference PRJNA498010.

2 Supplementary Figures and Tables

2.1 Supplementary Figures

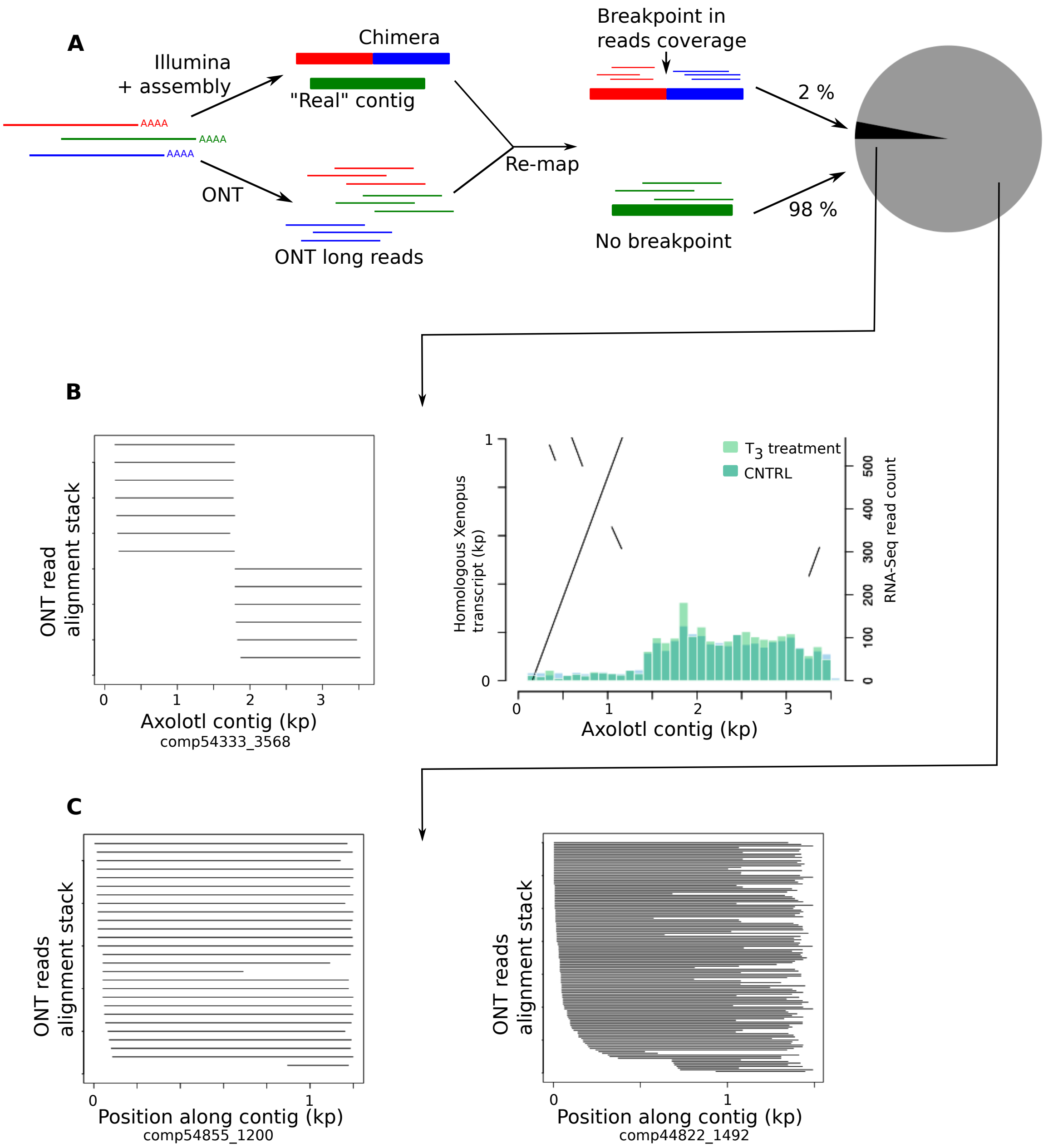

Supplementary Figure 1: Assessment of chimerism level in Axolotl transcriptome assembly.
A) RNA samples sequenced for transcriptome assembly were re-sequenced with ONT plateform. Breakpoint in reads coverage after mapping of the long ONT reads identifies chimeras.
B) Alignment stack of ONT reads on contig comp54333_3568, a chimeric contig. Notice that no read encompass the central region. This result is confirmed with a dot-plot analysis of the Axolotl contigs against the homologous sequence found in *X. tropicalis*. Notice that the transcript can be split in two halves, based on shared similarities (diagonals) and expression level. Histograms at the bottom of each dotplot correspond to the local read density of RNASeq reads (Dark cyan: control; light cyan: T_3 treatment) mapped on the transcripts. This contig represent genes which are not differentially regulated. C) Random examples of Illumina contigs well supported by ONT reads.

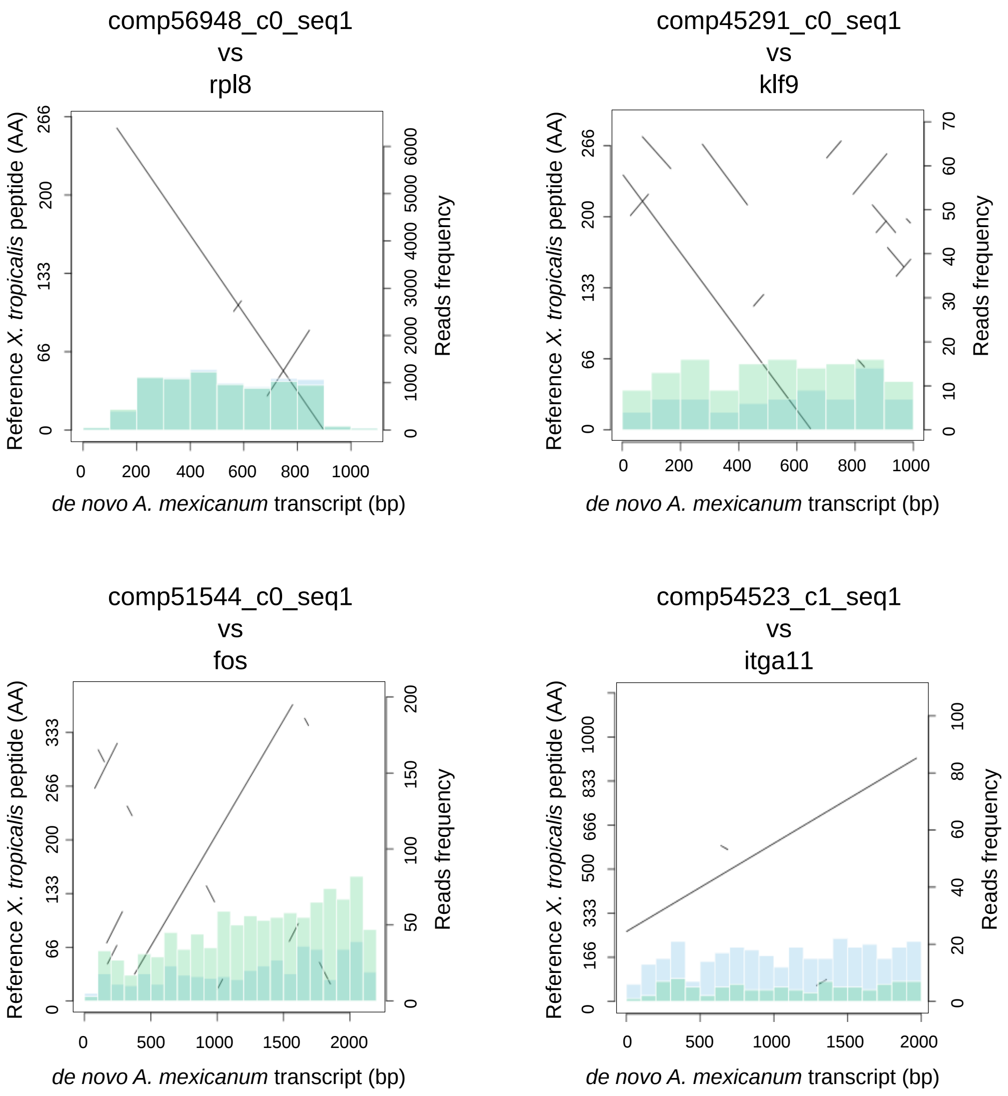

Supplementary Figure 2: Orthologous assembled transcripts align well between Axolotl and *X. tropicalis.* Sequence alignment between reference (*X. tropicalis*) transcripts sequence and *de novo* assembly of Axolotl transcripts. Illustrative examples of BLASTX alignments displayed as dot-plots. Alignments based on BLOSUM 45 scoring matrix. Histograms at the bottom of each dotplot correspond to the local read density of RNASeq reads (Dark cyan: control; light cyan: T_3 treatment) mapped on the transcripts. AA: amino-acids.

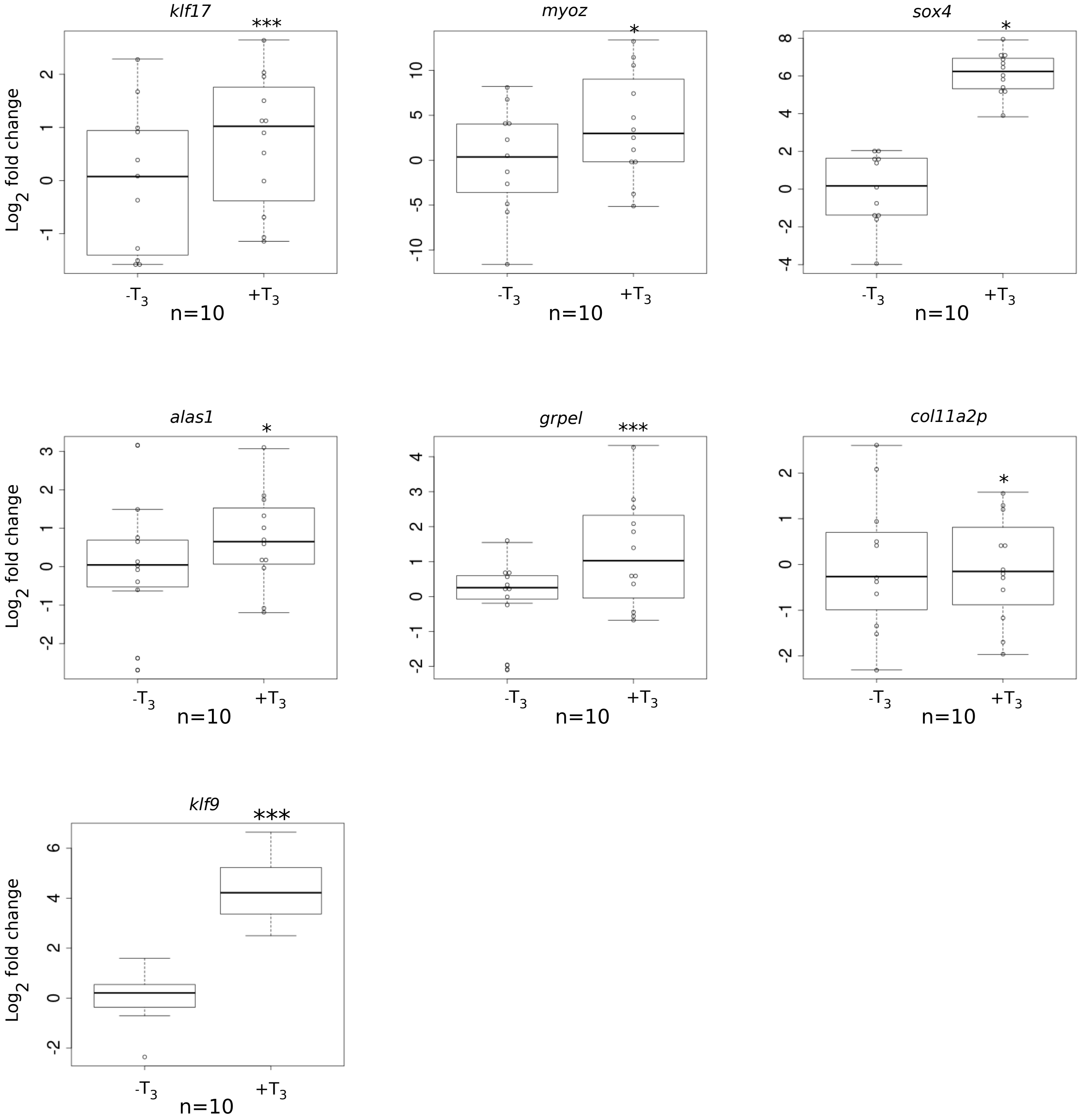

Supplementary Figure 3: RT-qPCR validation of the differential expression status, based on RNA-Seq data after T_3 treatment at HTP. Statistical significance (Mann-Whitney test) with * p<=0.05, ** p<=0.01, *** p<=0.001.

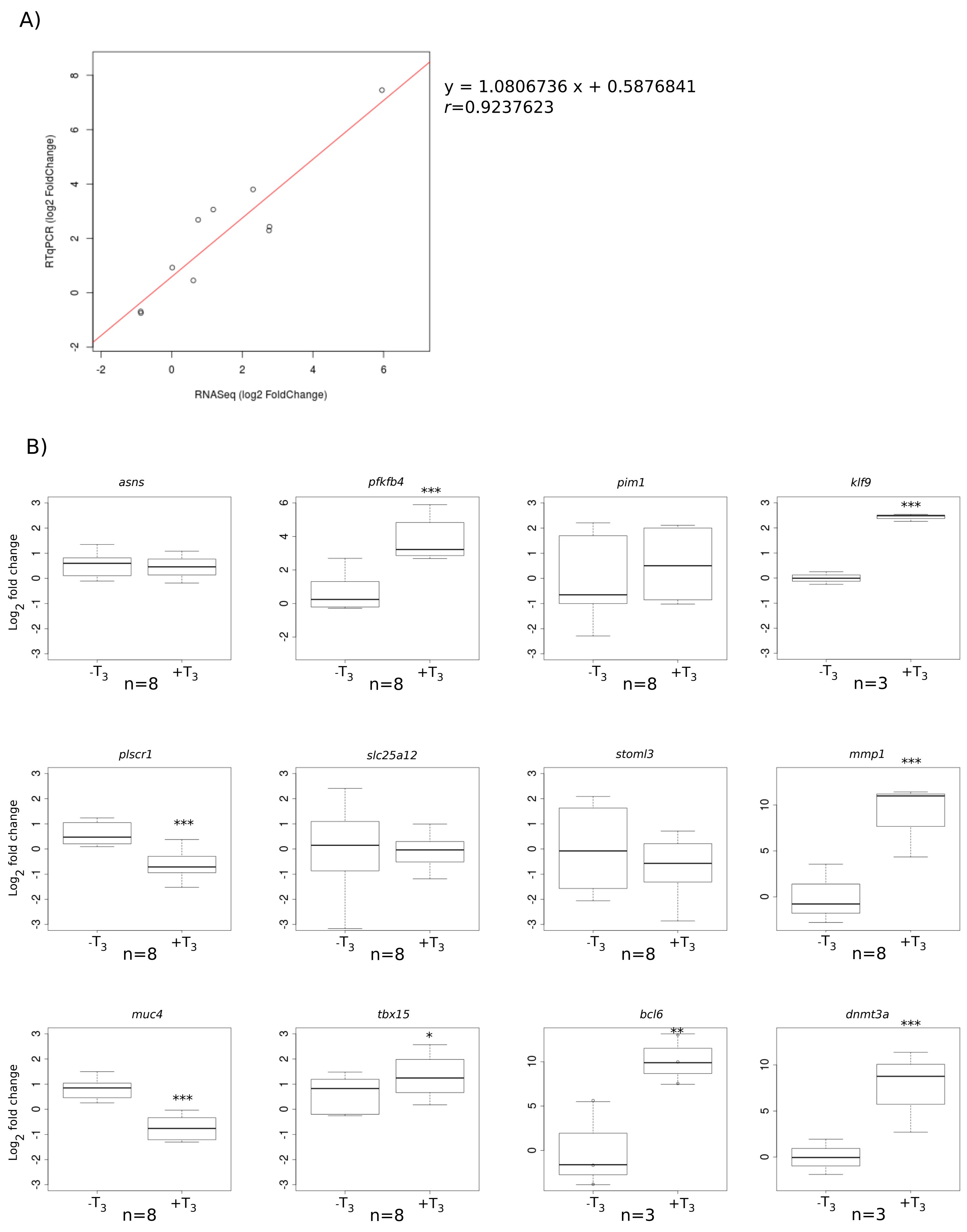

Supplementary Figure 4: Validation of the differential expression status of selected genes, in *X. tropicalis*. A) Relationship between gene expression change measured by RNA-Seq (see main text) and RT-qPCR (below). Red: Correlation curve. B) Measure of gene expression by RT-qPCR, with n=3 to 8 biological replicates. Statistical significance (Mann-Whitney test) with * $p<=0.05$, ** $p<=0.01$, *** $p<=0.001$.
